# Failure to Fuse Shut Eyelids, a Novel Unique Sign in Affected Fetus with Homozygous PPP1R13L Pathogenic Variant—A Case Report and Review of the Literature

**DOI:** 10.3390/ijms27156991

**Published:** 2026-08-04

**Authors:** Zaher Shalata, Hila Mintz, Sami Haddad, Khaled Osman, Mohammad Mahroum, Yarin Hadid, Adel Shalata

**Affiliations:** 1 S.B.A Rihana Medical Center, Sakhnin 30810, Israel; zaher.shalata@gmail.com; 2 The Simon Winter Institute for Human Genetics, Bnai Zion Medical Center, Haifa 31048, Israel or hila.mintz@sinaihealth.ca (H.M.); sami248@bezeqint.net (S.H.); khaled.osman@b-zion.org.il (K.O.); mohammed.mahroum@b-zion.org.il (M.M.); yarin.hadid@b-zion.org.il (Y.H.); 3 Obstetrics-Gynecology Ultrasound Unit, Bnai-Zion Medical Center, Haifa 31048, Israel; 4 The Ruth and Bruce Rappaport Faculty of Medicine, Technion, Haifa 32000, Israel

**Keywords:** sonography, fetus, open-eye, eyelid development, *PPP1R13L*, iASPP, NF-κB, SMAD signaling

## Abstract

Prompted by a fetus with a homozygous *PPP1R13L* pathogenic variant and persistent open eyelids on sonography, suggesting fusion failure, this study marks the first prenatal human description linking *PPP1R13L* to eyelid closure, mirroring waved with open eyelids 2(woe2) and waved 3 (wa3) mouse models. Eyelid morphogenesis involves complex epidermal–dermal interactions. We reviewed the literature to understand embryonic/fetal development and identify molecular pathways crucial for proper eyelid morphology. Known signaling pathways in eyelid closure and reopening include NF-κB, p53, Wnt, TGF-β, BMP4, MAPK, SMAD, ECM–receptor interaction, and integrin signaling. Open-eye phenotypes are associated with pathogenic variants affecting the Wnt/β-catenin pathway, p63, and the TGFβ family. Notably, SMAD molecules are common to all these pathways. Eyelid closure seems regulated by SMAD protein activation in the supraorbital epithelium and underlying mesenchyme, promoting cell proliferation and adhesion. Physiological SMAD1 downregulation is crucial for eyelid reopening. Given SMAD signaling’s importance in development, its disruption likely impacts eyelid closure or reopening.

## 1. Introduction

Recent data from the Medikeye study and the CDC indicate that congenital eye anomalies (CEAs) occur in approximately 3 to 4.5 out of every 10,000 births. Congenital cataracts are the most frequent type, accounting for about 31% of CEA cases, followed by anophthalmia/microphthalmia at roughly 24%. Coloboma is another common CEA, frequently associated with genetic syndromes or factors that remain unidentified. The prevalence of CEAs can differ depending on the geographic region and the diagnostic techniques employed. The comprehensive data generated by these studies underscore the critical need for early detection of CEAs to prevent significant vision impairment [1,2].

The development of the eyes and eyelids proceeds in a precise, sequential manner, where disruptions in earlier stages can impede subsequent steps [3]. Eyelid morphogenesis, in particular, is a complex and dynamic process that relies on intricate interactions between the epidermis and dermis [4]. This stage requires the coordinated activity of numerous signaling molecules and pathways, highlighting the importance of both intraepithelial and epithelial–mesenchymal interactions for proper eyelid formation [5,6,7,8,9]. Furthermore, the development of the eyelid involves the collaboration of the surface ectoderm with secondary mesenchyme, which arises from the invasion of the mesoderm by cranial neural crest cells [10].

Important timeline steps in eyelid development occur around 6–7 weeks of gestational age (GA), when the initial stages of eyelid formation are marked by the appearance of small grooves in the surface ectoderm above and below the developing eye [11,12,13]. This process involves the proliferation and migration of epithelial cells, leading to deepening and growth of the upper and lower eyelid folds [11,12,13]. And by approximately the 8th week (GA), the leading edges of the developing eyelid folds (the marginal epithelium) make contact across the cornea. The eyelids then fuse shut around 10 weeks GA, typically progressing from the outer corner (lateral canthus) towards the inner corner in a zipper-like fashion [14]. This closure is maintained for several weeks. Eyelid separation commences between 20 and 24 weeks GA, starting from the nasal side and progressing towards the temporal side [14].

After the eyelids separate, the fetus begins developing the blink reflex as its nervous system matures. While general eye movements and sensitivity to light can occur earlier (around 22–24 weeks), the ability to open the eyelids and consistently perform a blinking action typically emerges and becomes more reliable between 24 and 28 weeks, marking a final stage of eye maturation before birth [14,15]. Eye blinks are crucial for neurological maturation and are an important aspect of facial expression. Recent research has extensively explored the perceptual consequences and neurophysiology of eye blinks [15].

A 2003 study found that approximately 89% of healthy fetuses (33–42 weeks gestational age) blinked, with an average frequency of 6.2 blinks per hour. Vibroacoustic stimulation significantly increased blinking to an average of 15.3 blinks per hour in a subset of these fetuses [16]. Newborns blink less frequently (6.2 blinks/minute) than preschool children (8.0 blinks/minute) and exhibit slower eyelid movements [17]. Adult blink rate is approximately 15 blinks/minute [15].

In cooperation with Falik-Zaccai TC, we reported on a *PPP1R13L* gene pathogenic variant encoding the iASPP (inhibitor of Apoptosis-Stimulating Protein of P53) protein, associated with arrhythmogenic cardiomyopathy with variable ectodermal abnormalities and multiple congenital anomalies/dysmorphic syndrome. Affected individuals develop severe dilated cardiomyopathy (DCM), which can lead to death, and exhibit cutaneous manifestations, including woolly or wiry hair, wedged teeth, xerotic skin, dystrophic nails, cleft palate, and corneal abnormalities [18].

In this case report, we describe a distinctive sonographic finding: open eyelids in both eyes, initially observed at 15 weeks’ gestational age (GA) in a fetus of a consanguineous young couple (the parents of patient Y.M, (VI4) in the Falik-Zaccai TC publication). Prenatal diagnosis by amniocentesis was performed, and the fetus was homozygous for a *PPP1R13L*-related disease pathogenic variant [18], and the pregnancy was terminated at 20 weeks GA. Fetal ultrasound follow-up revealed continuously opened eyelids, suggesting a failure of eyelid fusion. Here, we describe our findings in detail, review the relevant literature, and discuss the involvement of *PPP1R13L*, alongside several other genes, and their signaling pathways known to be crucial for proper eyelid embryofetal morphology.

## 2. Detailed Case Report

**Figure 1:**
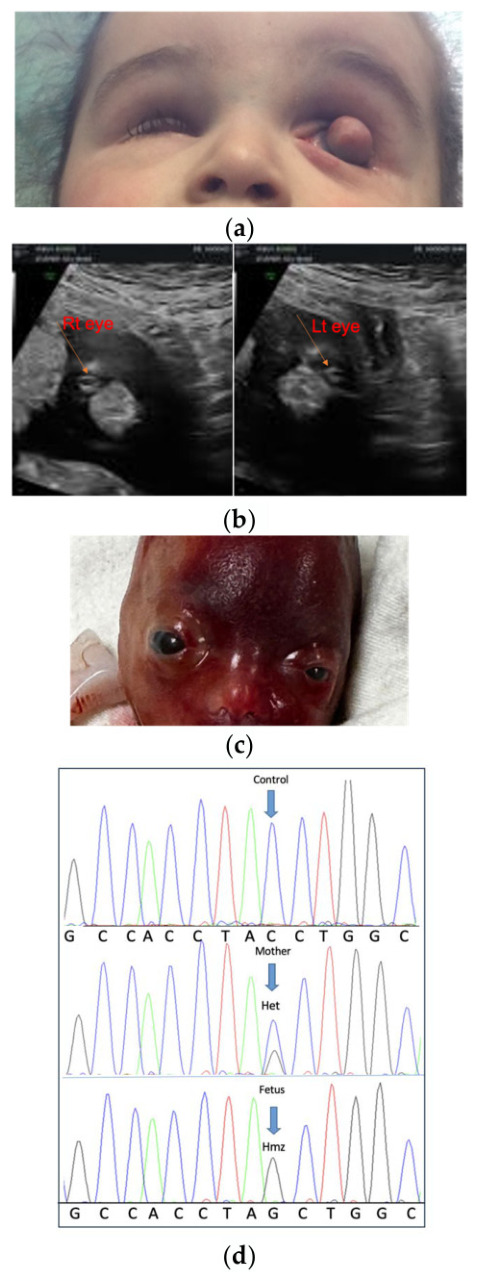
(**a**) Patient Y.M. (VI4) in the Falik-Zaccai TC 2017 publication [18]. Bilateral cloudy cornea and congenital corneal cyst (left eye). (**b**) Pictures and videos in the Supplementary Materials. Two video clips of prolonged sonography obviously demonstrate fixed bilateral open eyes. In both Video S1 (start from second 28) and Video S2 (start from second 2 to the end), both fetal eyes were continuously open, and they presented as echogenic lines. (**c**). Post-mortem fixed bilateral open eyes. (**d**) Picture of Sanger sequence for prenatal diagnosis, which includes the electropherogram of the affected fetus (**lower**) showing homozygous state for the pathogenic variant: *PPP1R13L*: c.2241C > G; p.Y747*, which is located in exon11 of transcript NM_006663 (chr19:45888827(hg19): c.2241C > G) compared with normal and heterozygous alleles in the control (**upper**) and the mother (**middle**), respectively.

We performed a routine, spontaneous pregnancy follow-up in a young, first-degree cousin couple (the parents of patient Y.M., VI4, as referenced in Falik-Zaccai TC, 2017 (Figure 1a) [18]. For fetal sonographic screening, we used the GE voluson E10. The nuchal translucency (NT) measurement was normal (1 mm). First-trimester biochemical screening also yielded normal results, indicating a low risk (1:6000) for Down syndrome. An ultrasound scan at 15 weeks’ gestation was otherwise unremarkable, apart from the unexpected observation of fixed open eyes bilaterally. One week later, a second ultrasound examination at Bnai-Zion Medical Center, which included a prolonged sonographic video inspection, definitively confirmed persistently fixed bilateral open eyes (Figure 1b (video in the Supplementary Materials) and Figure 1c).

Amniocentesis was performed at 18 weeks’ GA for prenatal diagnosis using direct DNA extracted from amniocytes. PCR amplification and Sanger sequencing of exon 11 (transcript NM_006663) of the *PPP1R13L* gene was conducted using standard methods (Sanger sequence technology) and primers described in the Falik-Zaccai TC 2017 manuscript, exhibiting homozygosity for the stop-gain pathogenic variant c.2241C > G, p.Tyr747Ter in *PPP1R13L*, disrupting the iASPP protein [18]. The pregnancy was terminated at 20 weeks’ GA. Although the couple declined a fetal pathological dissection study, they granted permission for photographs, as depicted in Figure 1c.

The ophthalmic phenotype can be seen very well in a photograph (Figure 1a) of patient Y.M. (VI:4) described in the Falik-Zaccai TC 2017 study [18]. She had bilateral cloudy corneas and a congenital corneal cyst, and she had no behavioral visual response. In a brain MRI of patient Y.M. (VI:4), there was evidence of distortion of the eyeball with a sand clock appearance, thin optic nerves, and cystic protrusion of the eyeball LT > RT. She died at 3.5 years of age secondary to cardiac complications.

To examine embryonic eyelid development, we conducted a targeted literature review focusing primarily on review articles we utilized PubMed (https://pubmed.ncbi.nlm.nih.gov) and Google Scholar (https://scholar.google.com). (Access dates were alongside the preparation and writing of this study intensively between January and April 2026 and 15–24 July 2026).

For molecular signaling pathway analysis, we used the following: 1. KEGG (Kyoto Encyclopedia of Genes and Genomes): (https://www.genome.jp/kegg); 2. Reactome: (https://reactome.org/); 3. BioCyc: (https://biocyc.org/).

For gene review, we used GeneCards, the human gene database (https://www.genecards.org/).

For protein–protein interactions we used the following: 1. STRING (Search Tool for the Retrieval of Interacting Genes/Proteins): (https://string-db.org/); 2. IntAct: (https://www.ebi.ac.uk/intact); 3. BioGRID (Biological General Repository for Interaction Datasets): (https://thebiogrid.org/). (Data searches and website access were conducted on a weekly basis from 15 January 2026 to 15 April 2026).

The eyelid anomalies were included within the subgroup of “congenital anomalies of the eyelids, lacrimal apparatus, and orbit” with a prevalence of 0.54 per 10,000 births [1]. Several OMIM entries and pathologic processes are known to affect the eyelids in direct and indirect manners. Hence, the eyelid phenotype and abnormality spectrum are wide, and the phenotype varies from minor changes in structure and shape (e.g., epicanthus, telecanthus, down-slanting palpebral fissures) to severe ones (e.g., cryptophthalmos and coloboma). We have summarized the major pathogenic mechanisms in brief in Table 1 to emphasize the different approaches used to describe these phenotypes. The phenotypes can be categorized as follows: structural abnormalities mostly confined to the orbit and eyelid; structural abnormalities involving the craniofacial region; eyelid involvement as part of systemic disease; diseases affecting the neuromuscular junction; congenital myopathies; and congenital ptosis and congenital orbital masses/lesions that involve the eyelid or affect its movement.

A reasonable estimate is that more than 50 genes are implicated in eyelid structural abnormalities; representative genes are depicted in Table 1. These include dominant ones such as *FOXL2* and *MYH14* genes, both of which are responsible for blepharophimosis-ptosis-epicanthus inversus syndrome (BPES). BPES is a classic example in which eyelid abnormalities, including small palpebral fissures, droopy eyelids, and epicanthal folds, are core features. In comparison, the *CHD7* gene is responsible for CHARGE syndrome, in which eyelid coloboma can be a feature along with other characteristic findings. Moreover, the *KMT2D* and *KDM6A* genes are involved in Kabuki syndrome, in which eyelid eversion is a reasonably common feature. *TCOF1*, *POLR1D*, *POLR1B* (dominant), and *POLR1C* or POLR1D (autosomal recessive) are responsible for Treacher Collins syndrome, in which lower eyelid coloboma is a classic feature. In addition, a mild eyelid phenotype is seen in Noonan syndrome caused by the *BRAF*, *KRAS*, *MAP2K1*, *MRAS*, *NRAS*, *PTPN11*, *RAF1*, *RASA2*, *RIT1*, *RRAS2*, *SOS1*, *SOS2*, and *LZTR1* genes, in which ptosis and an unusual eyelid shape can be seen. However, craniofacial syndromes that affect skull and facial bone development, including Apert, Crouzon (*FGFR2* gene), and Pfeiffer (*FGFR1*, *FGFR2* genes) syndromes, may have secondary effects on eyelid position and function.

Of note, eyelid abnormalities are common in copy number variation syndromes (i.e., aneuploidy, microdeletion, and microduplication), but are generally present with a mild phenotype. For example, in Down syndrome (Trisomy 21), upward-slanting palpebral fissures and epicanthal folds are frequently observed. Table 2 summarizes the primary ophthalmic findings in several microdeletion syndromes (including 22q11.2, 14q22q23, 3q26–3q27.2, 6p25, and 8q21.11) [19,20,21,22,23] and microduplication syndromes (including 22q11.2, 20q11.2, 3q29, 16p11.2, and Xq25) that are associated with eyelid abnormalities [24,25].

Despite the current lack of a complete understanding of the precise mechanisms of iASPP protein interactions and sequential molecular events during fetal eyelid development, this paragraph will attempt (at a theoretical level) to delineate the proteins, gleaned from the extensive list of *PPP1R13L* interactions (e.g., GeneCards), that are implicated in eyelid abnormalities and syndromes. We found six syndromic genes, including *SOX2* (Microphthalmia syndrome), which is present with microphthalmia, anophthalmia, clinical optic nerve hypoplasia, and coloboma; *CREBBP* and *EP300* (Rubinstein-Taybe syndrome 1 and 2), which are present with heavy high-arched eyebrows, long eyelashes, ptosis, epicanthal folds, nasolacrimal duct obstruction, strabismus, cataracts, glaucoma, coloboma, and down-slanting palpebral fissures; the CDH1 protein (BlepharoCheiloDontic syndrome-1 (BCDS1), which is present with hypertelorism, ectropion of lower eyelids, lagophthalmia (incomplete closure of eyelids), megaloblepharon, distichiasis (double row of eyelashes); the PPP1CB protein (Noonan syndrome), which is present with down-slanting palpebral fissures and ptosis; and finally, TP63, which is associated with two main relevant entities involved with eyelid structure, including “ankyloblepharon-ectodermal defects-cleft lip/palate” and ADULT syndrome (acro-dermato-ungual-lacrimal-tooth). For further theoretical evaluation and looking at eyelid reopening as an endpoint of proper development, we chose Fraser syndrome as an example. Fraser syndrome presents with a complex phenotype resulting from biallelic mutations in *FRAS1*, *FREM1*, and *FREM2* and manifests with cryptophthalmos, absent or malformed lacrimal glands, and hypertelorism [26]. Although a direct interaction between iASPP and the FRAS1 or FREM2 proteins has not been established yet, we assume that there is a potential indirect interaction. *FRAS1* encodes an extracellular matrix protein involved in regulating epidermal-basement membrane adhesion and organogenesis during development. Meanwhile, *FREM2* encodes an integral membrane protein containing numerous CSPG (chondroitin sulfate proteoglycan element) repeats and Calx-beta domains. This protein localizes to the basement membrane, forming a ternary complex that is important for epidermal–dermal interactions and the integrity of skin and renal epithelia [27]. Reviewing the FRAS1 protein interaction list, we identified 11 proteins that also interact with the iASPP protein, including CDH1, SOX2, TP53, TRIO, TRIM25, CTTN, HSPA9, JAG1, NOTCH1, NOTCH3, and RNF20 (https://www.genecards.org/ accessed on 15 January 2026). Of these, six genes are known to be syndromic (i.e., *JAG1*—Alagille syndrome; *CDH1*—BCDS1; *SOX2*—microphthalmia syndrome; *TRIO*—intellectual developmental disorder, autosomal dominant 44, with microcephaly; *HSPA9*—even-plus syndrome; *NOTCH1*—Adams-Oliver syndrome 5; and *NOTCH3*—cerebral arteriopathy, autosomal dominant, with subcortical infarcts and leukoencephalopathy 1). Of those, *JAG1*, *CDH1*, and *SOX2* are associated with major ophthalmic signs. Moreover, analysis of the FREM2 (FRASER second gene) encoded protein interaction list shows six proteins, including JAG1, NPHP1, NSD1, OBSCN, RPS3, and TFAP2A (https://www.genecards.org/ accessed on 20 March 2026). Only two, JAG1 (Alagille syndrome) and TFAP2A (Branchio-Oculo-Facial syndrome), are associated with major ophthalmic and orbital abnormalities. In the context of eyelid development, it remains an open question as to which key molecules are commonly shared across the various signaling pathways involved.

## 3. Discussion

To the best of our knowledge, this is the first reported case of fetal “open eyes” in the second trimester of pregnancy, diagnosed sonographically in a fetus harboring a homozygous familial pathogenic variant in the *PPP1R13L* gene. This finding definitively provides clear evidence of failed embryonic–fetal eyelid closure. The specific pathogenic variant described above is highly prevalent in a particular village in northern Israel due to a founder effect and a high rate of consanguineous marriages, leading to its inclusion in the national genetic screening panel.

Based on published literature and ClinVar, pathogenic variants in *PPP1R13L* are rare and mostly of a loss-of-function type. This includes 12 germline variants (8 frameshift, 3 nonsense, and 1 missense) [28].

Of note, the mouse strains waved with open eyelids 2 (woe2) and wa 3 both exhibit eyelids open at birth (normally closed) and wavy fur phenotypes. These strains harbor different spontaneous pathogenic variants in *PPP1R13L*, the mouse *PPP1R13L orthologous* gene, resulting in a complete loss of *PPP1R13L* function [29,30]. Immunohistological analysis during eye development in mice identified expression of *PPP1R13L* in the palpebral epidermis, palpebral and bulbar conjunctiva, corneal epithelium, and meibomian glands [29].

Our observation of open eyelids in the same nuclear family, including the fetus in the current study and patient Y.M (VI4) from the Falik-Zaccai TC 2017 publication [18], provides strong proof that *PPP1R13L* plays a central role in fetal eyelid closure in mammals. The data points directly to a fusion defect rather than an early separation, which aligns perfectly with the mouse models for the gene and its associated pathway’s function [29,30].

Clinical and histological analysis of adult woe2 eyes revealed severe corneal opacities, abnormalities of the anterior segment, and the absence of meibomian glands [29,30]. Consistent with these findings, our first patient presented with bilateral cloudy corneas and a congenital corneal cyst (Figure 1a). Furthermore, a brain MRI of Y.M. (VI4) showed distortion of the eyeball with a “sand clock” appearance, thin optic nerves, and cystic protrusion of the eyeball. However, we think that the additional postnatal findings involving the eyes (cloudy cornea, corneal cyst, lack of visual response, and so on) are likely a “sequence” or “secondary to” rather than separate primary defects of the eye, and most probably a consequence of the prolonged exposure of the developing eye to amniotic fluid due to the failed fusion and the lack of eyelid protection of the developing structures.

Eyelid development during embryogenesis is a sophisticated process involving coordinated communication between the surface ectoderm and underlying mesenchymal tissues. In both mice and humans, eyelids develop and fuse before birth, though they open at different times: prenatally in humans and postnatally in mice. In mice, growth factor Wnt signaling pathways are essential for the proper development of ocular tissues, specifically in driving eyelid growth by facilitating cell proliferation in both the supraorbital epithelium and the underlying mesenchyme [31]. Studies show that β-catenin disruption within the supraorbital mesenchyme abolishes eyelid development. Similarly, blocking Wnt secretion via the Wntless (Wls) deletion in the supraorbital epithelium results in defective eyelid formation, indicating that epithelial Wnt ligands are responsible for driving mesenchymal β-catenin signaling [31]. Furthermore, the deletion of p63 leads to hypoplastic eyelids and causes a decrease in Wnt ligand expression within the epithelium, suggesting that p63 partially governs the regulation of these Wnt ligands during development [31]. Moreover, FGF10, TGF-α, Activin B, and HB-EGF stimulate cell migration during this process, influencing downstream signaling pathways such as BMP4, ERK, and JNK/c-JUN [32,33,34,35]. Of note, TGFβ signaling pathway activation is a critical signaling pathway in eyelid fusion [36,37]. TGFβ ligands (like TGFβ1, TGFβ2, and TGFβ3) and their receptors are expressed in the developing eyelids, and activation of the TGFβ signaling pathway promotes epithelial remodeling, ECM deposition, and mesenchymal integration [36,37]. Thus, blocking TGFβ signaling can disrupt eyelid fusion in animal models, demonstrating its crucial role and importance [36,37].

Moreover, eyelid fusion itself can be inhibited and regulated by mechanisms like the Wnt/β-catenin pathway [38,39]. Conversely, eyelid opening relies on activation of the BMP/Smad signaling system [40]. Disruptions in these carefully orchestrated molecular pathways can lead to various human genetic disorders affecting eyelid development [41].

The *PPP1R13L* gene encodes iASPP (inhibitor of Apoptosis-Stimulating Protein of P53), an evolutionarily conserved ASPP family member crucial for regulating apoptosis and transcription [42]. iASPP achieves this regulation through interactions with NF-kappa-B, p53/TP53, and TP63 proteins [35,43].

**Figure 2:**
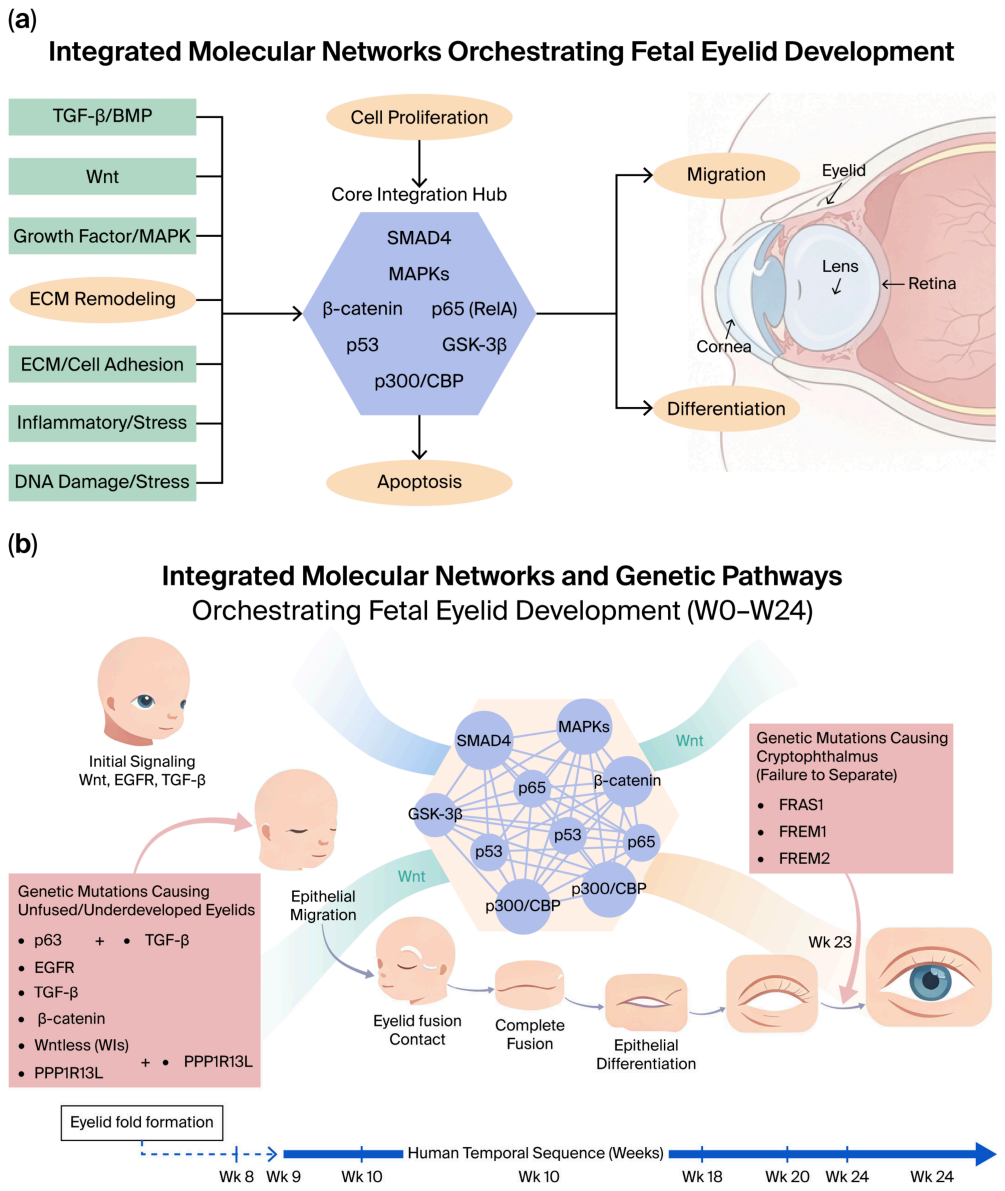
(**a**,**b**) This illustration, generated by the Nano Banana 2 Lite Machine Learning Model, depicts and simplifies (**a**) the stages of eyelid development (including fusion and separation) and (**b**) integrates them into a timeline axis. The cross-talk and integration of various pathways converge on a Core Integration Hub of common molecules, which include SMAD4 (Co-Smad), MAPKs (kinase cascade), β-catenin (TF), p65 (RelA) (TF), p53 (tumor suppressor/TF), GSK-3β (kinase), and p300/CBP (co-activator). This convergence ultimately leads to fine-tuned cellular responses. The images above highlight both robustness, where multiple inputs can influence a single critical step, and complexity, where shared nodes contribute to the intricate regulatory networks required for precise tissue development.

The signaling pathways critical for eyelid development—including NF-κB, p53, Wnt, TGF-β, BMP4, MAPK, SMAD, ECM–receptor interaction, and integrin signaling—are evidently heavily interconnected, operating as a complex network rather than isolated cascades [31,44] (Table 3, Figure 2). These pathways generally share key molecules that cross-regulate, activate, or inhibit one another, particularly during development, immune response, and cancer progression [44]. Our review of the literature identified numerous landmark studies exploring the molecular mechanisms of eyelid morphogenesis, both directly and indirectly, all of which underscore the crucial interplay of these pathways and molecules in mammalian eyelid development (primarily in mouse models, which are highly relevant to humans).

This paragraph will briefly outline the main pathways and molecules (Table 3, Figure 2) involved in eyelid development, focusing on their roles in eyelid formation, fusion, and separation [8,9,13,45].

The primary pathways include:

1. SMADs (SMAD2/3, SMAD1/5/8, SMAD4) are central to eyelid development, playing crucial roles in both eyelid closing and reopening (Table 3, Figure 2). Evidence for this includes studies showing that TGF-βRII mutations cause non-fusion or delayed fusion of eyelids [45,46,47]. Additionally, the deletion of SMAD4 (a common Smad for both TGF-β and BMP pathways) in mice leads to eyelid non-fusion and corneal abnormalities [46,48].
2. The Wnt pathway (involving β-catenin, GSK-3β, and p300/CBP) is essential for epithelial–mesenchymal interactions, placode formation, hair follicle (eyelash) development, and epidermal differentiation. β-catenin serves as the key effector, with its stability regulated by GSK-3β, and p300/CBP act as co-activators for β-catenin [21,29,45]. While its role is broad for skin, β-catenin has a central role in hair follicle (and thus eyelash) development and overall epidermal appendage formation, which is crucial for eyelid structure. Specifically, studies directly link β-catenin to eyelid development, showing that its conditional deletion in the epidermis causes severe eyelid malformations and non-fusion [31].
3. The MAPK pathway, encompassing ERK, JNK, and p38, is activated downstream of growth factor receptors like EGFR and regulates crucial cellular processes such as proliferation, survival, and differentiation. This pathway is essential for eyelid fusion [49]. For instance, EGFR is critical for proper eyelid development and fusion; its signaling primarily utilizes the MAPK cascade (ERK, JNK, p38) to drive the epithelial proliferation and migration required for eyelid formation and subsequent separation [50]. The observation that EGFR loss results in an “eyes open at birth” phenotype—indicating a failure of eyelid fusion—directly implicates EGFR and its downstream MAPK signaling in this vital developmental process.
4. NF-κB: While prominently associated with inflammation and immunity, the NF-κB pathway, including p65/RelA and IKKs, also plays crucial developmental roles in cell survival, proliferation, and differentiation, particularly within epithelia [51]. p65 (RelA) serves as a key transcriptional component, critical for NF-κB’s broad developmental functions, which encompass cell proliferation and survival essential for tissue growth, including that of the eyelid. The activation of p65 by upstream IKKα/β kinases links the core NF-κB pathway to proper eyelid development and fusion, with dysregulation known to lead to specific eyelid fusion defects [52].
5. The p53 pathway, renowned as the “guardian of the genome,” primarily induces cell cycle arrest or apoptosis in response to cellular stress and damage. In a broader developmental context, p53 plays a crucial role in tissue sculpting through the regulation of programmed cell death. While programmed cell death is essential for processes such as eyelid separation and opening, p53’s direct involvement in this specific eyelid apoptosis appears more indirect or context-dependent. Instead of being a primary, constitutive inducer for all eyelid sculpting events, its role is likely amplified under specific stress conditions or serves to fine-tune developmental apoptotic pathways, a pattern observed in various other tissues [53].
6. The integrin/ECM–receptor pathway involves integrins, transmembrane receptors critical for mediating cell-extracellular matrix (ECM) adhesion and crucial bidirectional signaling. These interactions profoundly influence cell migration, proliferation, differentiation, and overall tissue architecture. In the context of eyelid development and fusion, integrins are essential for structural integrity and dynamic tissue remodeling. While established for their role in wound healing [54]—a process that shares fundamental mechanisms with developmental fusion—integrins link the ECM to intracellular signaling pathways, including MAPK cascades. This regulation of cell migration and proliferation is vital for the precise formation and subsequent fusion of the eyelids. Specifically, the integrin subunit β1 plays a crucial role in epidermal differentiation, which is indispensable for forming the complex stratified epithelium of the eyelids and facilitating proper cell-matrix interactions during fusion [55].
7. Co-activators p300 and CBP are histone acetyltransferases (HATs) that serve as crucial co-activators for a wide array of transcription factors, including β-catenin, p65, p53, and Smad proteins. By bridging these transcription factors to the basal transcriptional machinery, CBP/p300 effectively enhances gene expression [56]. While not specific to eyelid development, their indispensable role in modulating nearly all critical transcription factors and pathways relevant to development (e.g., Wnt, p53, Smads, NF-κB, MAPK targets) makes them indirect but essential players in every developmental process, including eyelid formation and fusion. Their precise contribution to eyelid development is thus context-dependent, determined by the specific transcription factors they co-activate.

## 4. Conclusions

Pathogenic variants in *PPP1R13L* result in defective eyelid closure in both humans and mice. In this context of eyelid development, the iASPP protein plays a crucial role in regulating cellular apoptosis and transcription [42]. iASPP achieves this regulation through interactions with several key proteins involved in eyelid fusion, such as NF-κB, p53/TP53, and TP63 (Table 3, Figure 2) [35,43]. Concurrently, the process of eyelid closure appears to be critically regulated by SMAD protein activation. Specifically, SMAD activation in the supraorbital epithelium and underlying mesenchyme promotes essential cell proliferation and adhesion. Given the vital role of SMAD signaling in orchestrating these developmental processes, any disruption to this axis would predictably impair proper eyelid closure or reopening. We propose that FRAS1, FREM1, and FREM2 modulate SMAD1 activity by differentially regulating BMP4 (a SMAD1 activator) and GDF11 (a SMAD1 repressor). Specifically, FRAS1/FREM1/FREM2-mediated repression of BMP4 and activation of GDF11 is hypothesized to drive the crucial downregulation of SMAD1 required for proper eyelid development. Consequently, loss-of-function mutations in FRAS1, FREM1, or FREM2, as seen in Fraser syndrome, would disrupt this fine-tuned regulatory balance, potentially resulting in cryptophthalmos. This model posits that these upstream pathways converge to modulate SMAD signaling during eyelid development. Therefore, future research should prioritize investigating altered SMAD activity as a key downstream mechanism contributing to the observed open-eye phenotype. Simultaneously, validating this model necessitates a thorough investigation of pathway-specific effects and a comprehensive characterization of the molecular interactions between FRAS1/FREM1/FREM2, BMP4/GDF11, and SMAD1 during eyelid development.

## Figures and Tables

**Table 1:** Eyelid phenotype subgroups in brief.

Group	Dominant Phenotype When Applicable	HPO	HPO-Human Phenotype Ontology (jax.org)	Notable Representing Syndromes and Some Affiliated Genes or Locations		Reference
Structural abnormalities mostly confined to the orbit and eyelid	Blepharophimosis	HP:0000581	129	Autosomal-dominant blepharophimosis-ptosis-epicanthus inversus syndrome (BPES)	FOXL2	PMID: 28280295
	Cryptophthalmos	HP:0001126	6	Cryptophthalmos, unilateral or bilateral, isolated	FREM2	OMIM-#123570
				Microphthalmia/coloboma and skeletal dysplasia syndrome	MAB21L2	Human Phenotype Ontology (jax.org)
	Eyelid aplasia or hypoplasis aplasia/hypoplasia of the eyelid	HP:0011226	44	Neu-Laxova syndrome	PHGDH, PSAT1	PMID: 35885441
				Ablepharon-macrostomia syndrome	TWIST2	PMID: 21595001
Congenital ptosis	Ptosis	HP:0000508	736	Ptosis, congenital 1	*ZFHX4*	PMID: 28280295
				Ptosis, hereditary congenital 2	XQ24–Q27.1	
				Duane syndrome	MAFB	Ptosis, Hereditary Congenital 2-MalaCards
				3MC syndrome	COLEC10	
Structural abnormalities involving the craniofacial region	Craniosynostosis	HP:0001363	180	Non-syndromic craniosynostosis	Ras/ERK	PMID: 28808027
	Encephalocele	HP:0002084	120	Frontorhiny	ALX3	PMID: 25126130
						PMID: 19409524
Eyelid involvement as part of systemic disease				Treacher Collins syndrome	*TCOF1*, *POLR1D*, *PLR1C* and *POLR1B*	PMID: 34573374
				Goldenhar syndrome	unknown etiology	PMID: 30134872
				Fraser syndrome	FRAS1 FREM1 FREM2	PMID: 35791156
						OMIM # 219000
				Gordon syndrome	PIEZO2	OMIM # 114300
				Crouzon syndrome	FGFR2	OMIM # 123500
				Blepharophimosis-ptosis-intellectual disability syndrome	UBE3B	PMID: 28280295
				Lymphedema-distichiasis-syndrome	*FOX2*	PMID: 28280295
				Noonan syndrome	KRAS and multiple affiliated genes)	PMID: 29948256
				Waardenburg syndrome subtypes	PAX3, MITF, SOX10	PMID: 36620710
				46, XX sex reversal 5	*NR2F2*	Blepharophimosis, Ptosis, and Epicanthus Inversus Syndrome-GeneReviews^®^-NCBI Bookshelf (nih.gov)
				Say–Barber–Biesecker variant of Ohdo syndrome	KAT6B	
Disease affecting the neuromuscular junction (NMJ)				Möbius syndrome type 2	3q21–q22	OMIM **#** 601471
						PMID: 25633065
				Congenital myasthenic syndrome 9	MUSK	OMIM # 616325
				Other congenital myasthenic syndromes	CHRNE, ALG14	PMID: 28280295
Congenital myopathies				Many congenital muscular diseases may be accompanied by congenital ptosis with or without additional ocular anomalies, including congenital myopathies, mitochondrial myopathies, myotonic syndromes, muscular dystrophies, and other myopathies	Multiple affiliated genes	PMID: 35049410
				In congenital fibrosis of the extraocular muscles (CFEOM) syndromes (1 + 2 + 3)	KIF21A, PHOX2A/ARIX	PMID: 28280295
Congenital orbital masses/lesions that involve the eyelid or affect its movement Group structural abnormalities mostly confined to the orbit and eyelid Congenital ptosis structural abnormalities involving the craniofacial region	Teratoma	HP:0009792	11	Sotos syndrome	NSD1	PMID: 34027878
						PMID: 28695872
	Periorbital dermoid cyst	HP:0030668	1	Otofaciocervical syndrome 2	PAX1	PMID: 34750318
	Peripheral primitive neuroectodermal neoplasm	HP:0030067	62	Mismatch repair cancer syndrome 1	MLH1	PMID: 34621833
	Aggressive infantile fibromatosis (desmoid tumor)	HP:6001034	7	Gardner syndrome	APC	
	Embryonal rhabdomyosarcoma	HP:0006743	4	Rhabdomyosarcoma, embryonal, 2	DICER1	
				Rhabdomyosarcoma 1	SLC22A18	
	Optic nerve sheath meningioma	HP:0500089	1	Neurofibromatosis, type II	NF2	PMID: 34621833
						DOI: 10.2147/IMCRJ.S82795
						(A congenital case report was not located in the literature)
	Periocular capillary hemangioma	HP:0500090	0			PMID: 28540013
	Granular cell tumor of the eyelid or orbit Dominant phenotype when applicable	An HP was not located for the phenotype		Noonan syndrome	Multiple genes	PMID: 34621833
						PMID: 19953625
						(A congenital case report was not located in the literature)

**Table 2:** Summary of copy number variation (microdeletion and microduplication) syndromes associated eyelid abnormalities and dysmorphic features.

Microdeletion Syndromes	Associated Eyelid Abnormalities	Microduplication Syndromes	Associated Eyelid Abnormalities
**16p11.2** PMID: 21465664 PMID: 38050025 PMID: 32373379	Down-slanting palpebral fissures; ptosis; sagging lateral upper eyelids; eyelid eversion; long eyelashes and prominent eyes	**22q11.2** PMID: 23972321 PMID: 30505980 PMID: 27108843	Highly variable, but commonly reported features include down-slanting palpebral fissures, ptosis (drooping eyelids), superior displacement of the eyebrows, hypertelorism (wide-set eyes), hooded eyelids, and, rarely, severe cases involving congenital entropion (inward-turning eyelids), ectropion (outward-turning eyelids), and corneal ulceration
**22q11.2** PMID: 17704945 PMID: 27182748 PMID: 26056486	Deep-set eyes; eyelid hooding, short (narrow) palpebral fissures	**20q11.2** PMID: 23704076 PMID: 30893560	Distinctive facial features can include epicanthus (skin folds at the inner corner of the eye), hypoplastic supraorbital ridges, and horizontal or down-slanting palpebral fissures
**14q22q23** PMID: 30268123 PMID: 22486322	Ptosis and hypertelorism	**3q29** PMID: 33039685 PMID: 19298871 PMID: 19287140	Ocular abnormalities can occur, with some cases involving a range of issues, including nystagmus, epicanthus, lagophthalmos (incomplete eyelid closure), entropion, and infantile glaucoma
**3q26.33**–**3q27.2** PMID: 23357683 PMID: 27525095 PMID: 24462885	Including narrow horizontal palpebral fissures, epicanthal folds, and ptosis (blepharophimosis sequence)	**16p11.2** PMID: 38605127 PMID: 24891046	Subtle and non-specific findings include down-slanting palpebral fissures, deep-set eyes, ptosis, hypertelorism, epicanthic folds, and subtle synophrys
**6p25** PMID: 15654696 PMID: 36941760 PMID: 15150541	Proptosis (bulging eyes) and down-slanting palpebral fissures	**Xq25** (**STAG2**) OMIM. #300387	Facial dysmorphism can include lower palpebral eversion; this microduplication has also been reported in a patient with eyelid myoclonia and absences (Jeavons syndrome)
**8q21.11** PMID: 21802062 PMID: 37039706 PMID: 36341706	Ptosis as part of facial dysmorphism		

**Table 3:** The key intersections of the signaling pathways mentioned in eyelid development: NF-κB, p53, Wnt, TGF-β, BMP4, MAPK, SMAD, ECM–receptor interaction, and integrin signaling. These key molecules function as hubs in various pathways that all contribute to the coordinated cellular behaviors needed for eyelid development, as illustrated in Figure 2.

Molecule/Complex =	Common Function	Primary Pathway	Shared Pathways
SMAD4	Transcription/Co-Smad	TGF-β/BMP	TGF-β, BMP4, SMAD
MAPKs	Kinase cascade	MAPK	MAPK, TGF-β, BMP, Int/ECM
β-catenin	Transcription factor	Wnt	Wnt, TGF-β/BMP (fibrosis)
p65 (RelA)	Transcription factor	NF-κB	NF-κB, p53, TGF-β
p53	Tumor suppressor	p53	p53, NF-κB, TGF-β/SMAD
GSK-3β	Kinase (destruction)	Wnt/MAPK	Wnt, MAPK, TGF-β
p300/CBP	Co-activator	General	Wnt, TGF-β, p53
Integrins	Receptor/adhesion	ECM-Rec	Integrin, MAPK, TGF-β

## Data Availability

Data is contained within the article or supplementary material.

## Supplementary Materials

The following supporting information can be downloaded at: https://www.mdpi.com/article/10.3390/ijms27156991/s1.

## Abbreviations

The following abbreviations are used in this manuscript:

CEAs	Congenital eye anomalies
GA	Gestational age
woe2	Waved with open eyelids 2
KEGG	Kyoto Encyclopedia of Genes and Genomes
