# Supplementary material for: Failure to Fuse Shut Eyelids, a Novel Unique Sign in Affected Fetus with Homozygous PPP1R13L Pathogenic Variant—A Case Report and Review of the Literature
Source: Int J Mol Sci. 2026 Aug 4;27(15):6991. doi: 10.3390/ijms27156991 (PMC13466727; doi:10.3390/ijms27156991)
Supplement: Supplementary file 1 [file ijms-27-06991-s001.zip › Supplementary Video S1 and S2_Caption.pdf]

Video S1 and S2. Two video clips of prolonged sonography obviously demonstrate fixed bilateral open eyes. In both clip1 (start from second 28) and clip2 (start from second 2 to the end), both fetal eyes were continuously open, and they presented as echogenic lines.
